# Supplementary figures and images for: Use of a highly specific kinase inhibitor for rapid, simple and precise synchronization of Plasmodium falciparum and Plasmodium knowlesi asexual blood-stage parasites
Source: PLoS One. 2020 Jul 16;15(7):e0235798. doi: 10.1371/journal.pone.0235798 (PMC7365400; doi:10.1371/journal.pone.0235798)

## Supp. figure 1

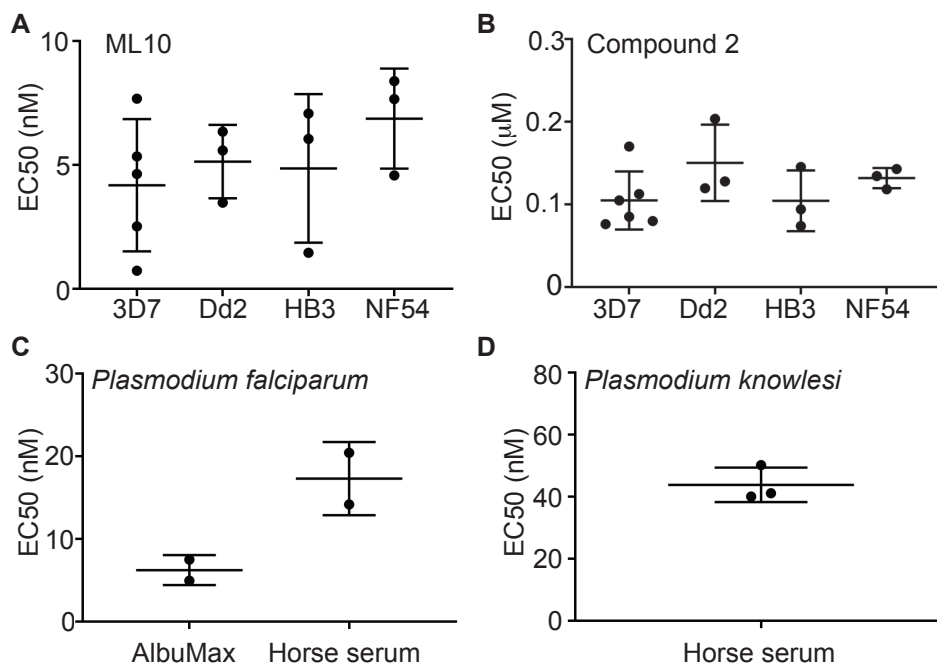

Supplement: S1 Fig — Mean EC50 values for P. falciparum strains 3D7, Dd2, HB3 and NF54 grown in standard culture conditions (0.5% AlbuMax) in the presence of ML10 (A) or Compound 2 (B). (C) Mean EC50 values obtained with P. falciparum strain 3D7 grown in standard culture conditions (0.5% AlbuMax) or in RPMI-1640 containing 0.5% AlbuMax and 10% horse serum. (D) Mean EC50 values obtained with P. knowlesi strain A1-H.1 grown in standard culture conditions using RPMI-1640 containing 0.5% AlbuMax and 10% horse serum. Differences in EC50 values between the P. falciparum strains were determined not to be significant using a Kruskal-Wallis one-way analysis of variance. Error bars are standard deviation of the calculated means. (PDF) [file pone.0235798.s001.pdf]

Supp. figure 2

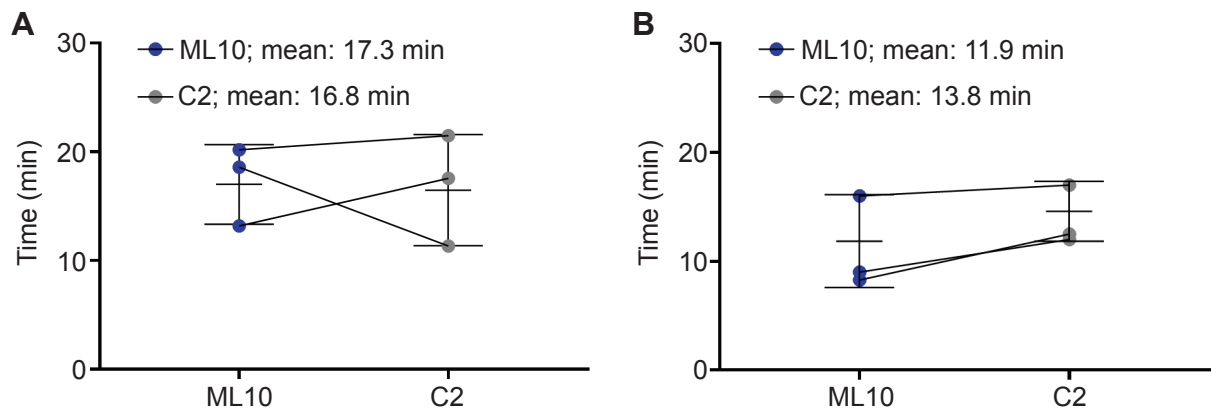

Supplement: S2 Fig — Quantification of the period until the first schizont egress event was detected (A) and the time required for egress of the majority of schizonts after egression of the first schizont (B). Values were obtained from the analysis of egress videos after removal of ML10 or Compound 2 from 3 independent experiments. No significant differences were detected using a paired t-test. (PDF) [file pone.0235798.s002.pdf]

Supp. figure 3

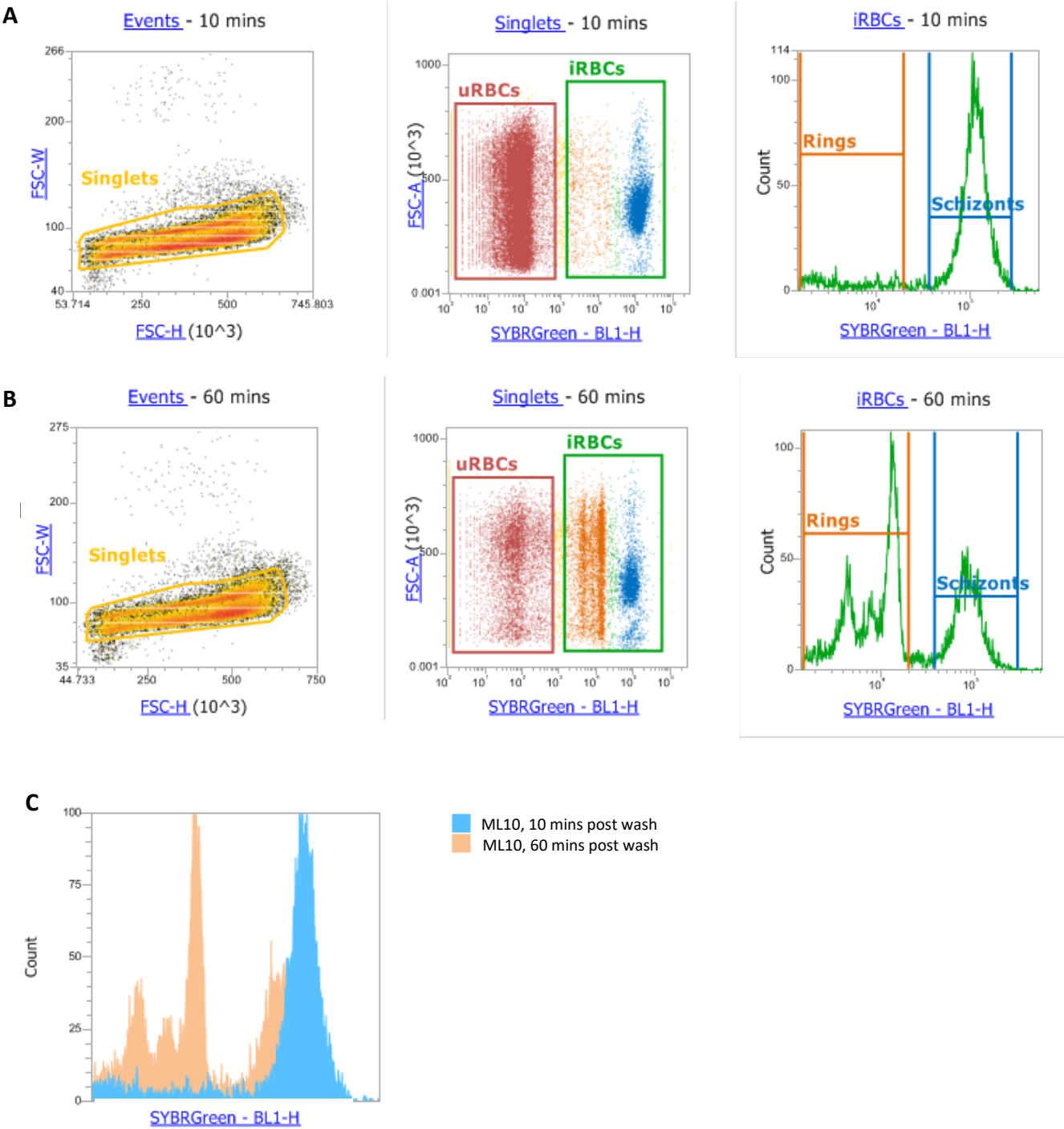

Supplement: S3 Fig — Representative gating strategies for the determination of parasitized erythrocytes stained with SYBR Green I nuclear staining and their respective ring and schizont stage, 10 minutes (A) and 60 minutes after (B) compound washout. (C) Overlay of histograms of the two time points emphasizing the differences in signal profiles between ring and schizont populations. (PDF) [file pone.0235798.s003.pdf]
